# Case Report: Usefulness of Drip Infusion Cholangiography With Computed Tomography for the Diagnosis of Biloma in a Dog

**DOI:** 10.3389/fvets.2022.854042

**Published:** 2022-03-07

**Authors:** Masahiro Tamura, Hiroshi Ohta, Nene Hasegawa, Kenji Hosoya, Mitsuyoshi Takiguchi

**Affiliations:** ^1^Department of Companion Animal Clinical Sciences, School of Veterinary Medicine, Rakuno Gakuen University, Ebetsu, Japan; ^2^Department of Veterinary Clinical Sciences, Graduate School of Veterinary Medicine, Hokkaido University, Sapporo, Japan; ^3^Veterinary Teaching Hospital, Graduate School of Veterinary Medicine, Hokkaido University, Sapporo, Japan

**Keywords:** biloma, cholangiography, computed tomography, drip infusion, DIC-CT, dog, gallbladder mucocele

## Abstract

Bilomas are encapsulated collections of bile outside or inside the biliary tract within the abdominal cavity. For diagnostic and therapeutic approaches, it is important to identify the origin of bile leaks from the biliary tract. This case report describes the usefulness of drip infusion cholangiography with computed tomography (DIC-CT) for detecting the site of bile leakage in a dog with biloma. A 10-year-old, castrated male Pomeranian dog was referred to our department for gastrointestinal signs. Abdominal ultrasonography detected gallbladder mucocele without evidence of defect on the wall and well-defined anechoic localized fluid accumulation around the right division of the liver. On the other hand, there was only a small amount of ascites in the abdominal cavity. The accumulated fluid collected through abdominocentesis had a bilirubin concentration of 11.4 mg/dl, which was more than twice as high as that in serum (0.4 mg/dl), but had absence of pyogenic bacteria. The DIC-CT with meglumine iotroxate showed two well-defined large fluid collections: one between right medial and lateral lobe and the other between the right lateral lobe and caudate process of caudate lobe. Three-dimensional DIC-CT views that the former was enhanced by the contrast agent and that it communicated with an intrahepatic bile duct of the right lateral lobe. Moreover, the DIC-CT images confirmed communication with each fluid collections. After 6 days of hospitalization, a decrease in the amount of accumulated fluid was confirmed, after which cholecystectomy was performed. The dog was discharged from the hospital without complications. No signs of bile leakage were observed on follow-up imaging on postoperative day 10. According to authors knowledge, this has been the first report to show that DIC-CT can be useful for determining the origin of bile leakage in dogs with bilomas.

## Introduction

Bilomas are encapsulated collections of bile outside or inside biliary tract within abdominal cavity (1). In humans, bilomas were reported to be results from abdominal trauma, spontaneous leakage of the biliary tree, or iatrogenic injury (2–5). Iatrogenic damage to the biliary tract is commonly associated with laparoscopic cholecystectomy (6). Bilomas in veterinary medicine has been described to be developed after surgery of intrahepatic portosystemic shunt (7), cholecystectomy (8), and open liver biopsy (9) and associated with trauma (10). It is important for diagnosis and therapeutic approaches to identify the site of bile leakage. Although ultrasonography is widely used for the screening of biliary system, it is difficult to evaluate the intrahepatic and common biliary duct in detail (11).

Drip infusion cholangiography with computed tomography (DIC-CT) using meglumine iotroxate is a commonly used imaging technique for the evaluation of the anatomy of the biliary tree and to confirm its patency in human. Recently, its usefulness was also reported in dogs with gallbladder mucocele and cats with cholelithiasis (12, 13). However, there was no report to evaluate the bile leakage using DIC-CT. In this case report, we described the usefulness of the DIC-CT for the detection of the site of bile leakage in a dog with biloma.

## Case Presentation

A 10-year-old, castrated male Pomeranian dog weighting 4.1 kg was referred to Hokkaido University Veterinary Teaching Hospital for examination due to history of lethargy, vomiting, and anorexia for 1–2 weeks. Several days prior to presentation, clinical signs had improved but mild gastrointestinal signs remained.

Physical examination at presentation was unremarkable, with the exception of slight abdominal discomfort, a heart and respiratory rate of 128 and 36/min, respectively, and a body temperature of 38.3 °C. Hematological abnormalities included mature neutrophilia (19.12 × 10^3^ neutrophils/μl; reference interval, 2.95 × 10^3^-11.64 × 10^3^ neutrophils/μl) and monocytosis (1.45 × 10^3^ monocytes/μl; reference interval, 0.16 × 10^3^-1.12 × 10^3^ monocytes/μl). Results of plasma biochemical analyses indicated elevated levels of total protein (7.8 g/dl; reference interval, 5.0–7.2 g/dl), blood urea nitrogen (39.1 mg/dl; reference interval, 9.2–29.2 mg/dl), and C-reactive protein (3.75 mg/dl; reference interval, 0.0–1.0 mg/dl); increased activity of alanine aminotransferase (174 IU/L; reference interval, 17–78 IU/L), alkaline phosphatase (>3,500 IU/L; reference interval, 47–254 IU/L), and gamma-glutamyl transpeptidase (124 IU/L; reference interval, 5–14 IU/L); and hyperphosphatemia (5.7 mg/dl; reference interval, 1.9–5.0 mg/dl) and hypercalcemia (12.8 mg/dl; reference interval, 9.3–12.1 mg/dl). Total bilirubin concentration was within reference limits (0.4 mg/dl; reference interval, 0.1–0.5 mg/dl). Prothrombin time and activated partial thromboplastin time was within normal limits, but fibrinogen concentration was increased (502 mg/dl; reference interval, 113–385 mg/dl). The dog showed low serum thyroxine (T4) but normal serum free T4 and thyroid stimulating hormone.

**Figure 1:**
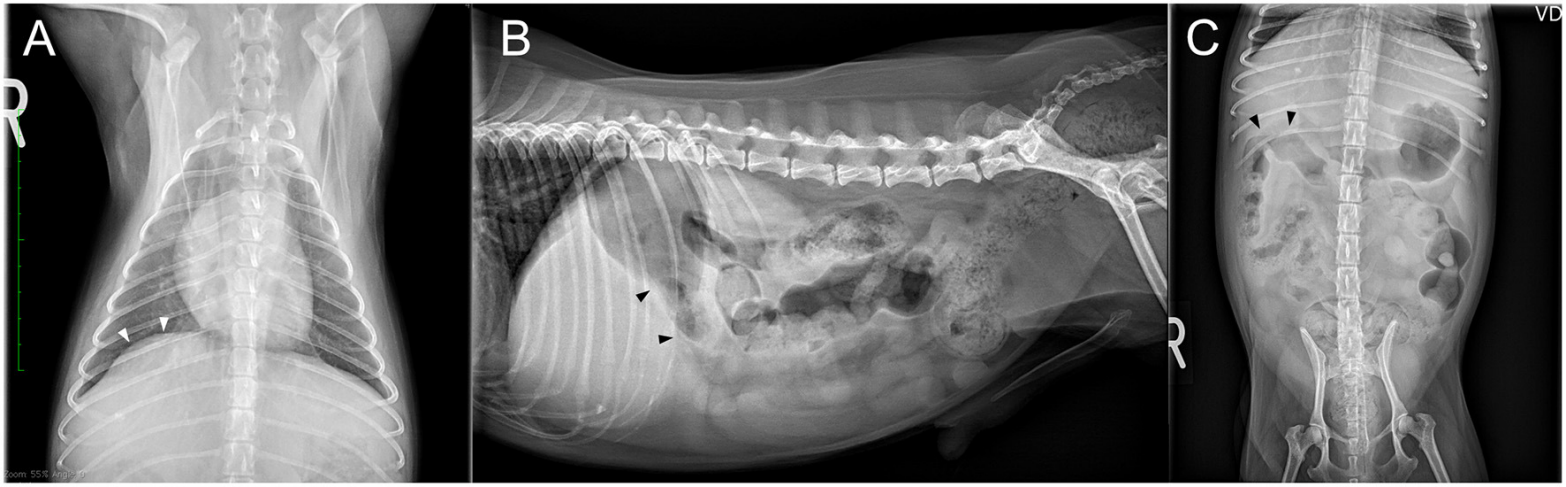
Three thoracic and abdominal radiographs obtained from a dog with biloma on initial presentation. The right crus of the diaphragm was found to have been cranially displaced by one intercostal space relative to the left crus in the ventrodorsal view of the thorax [**(A)**; white arrow-head]. The pylorus of stomach was displaced caudally in the lateral **(B)** and ventrodorsal **(C)** radiographic views of the abdomen (black arrow-head).

Radiography showed that the right crus of the diaphragm was cranially displaced by one intercostal space relative to the left crus in the ventrodorsal view of the thorax (Figure 1A). The caudal displacement of the pylorus of the stomach was suspected in the lateral and ventrodorsal radiographic view of the abdomen (Figures 1B,C). The remaining abdominal organs and thoracic radiographs were normal.

**Figure 2:**
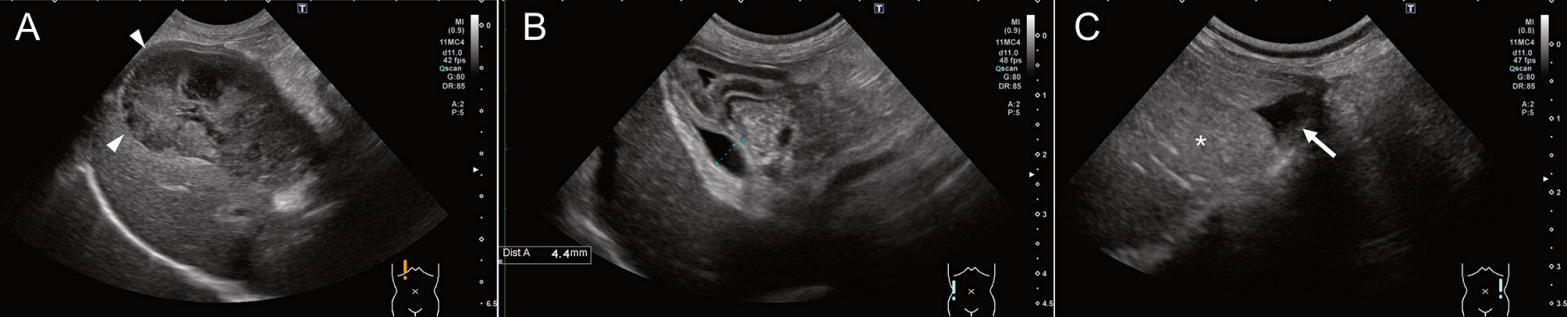
Ultrasonographic findings. **(A)** The gallbladder showed a kiwi-like pattern and stella combination with residual central echogenic bile (white arrow-head), indicating gallbladder mucocele. **(B)** The common biliary duct dilation without echogenic sediment or debris was observed (4.4 mm). **(C)** A small amount of ascites (white arrow) was detected around the spleen (asterisk).

**Figure 3:**
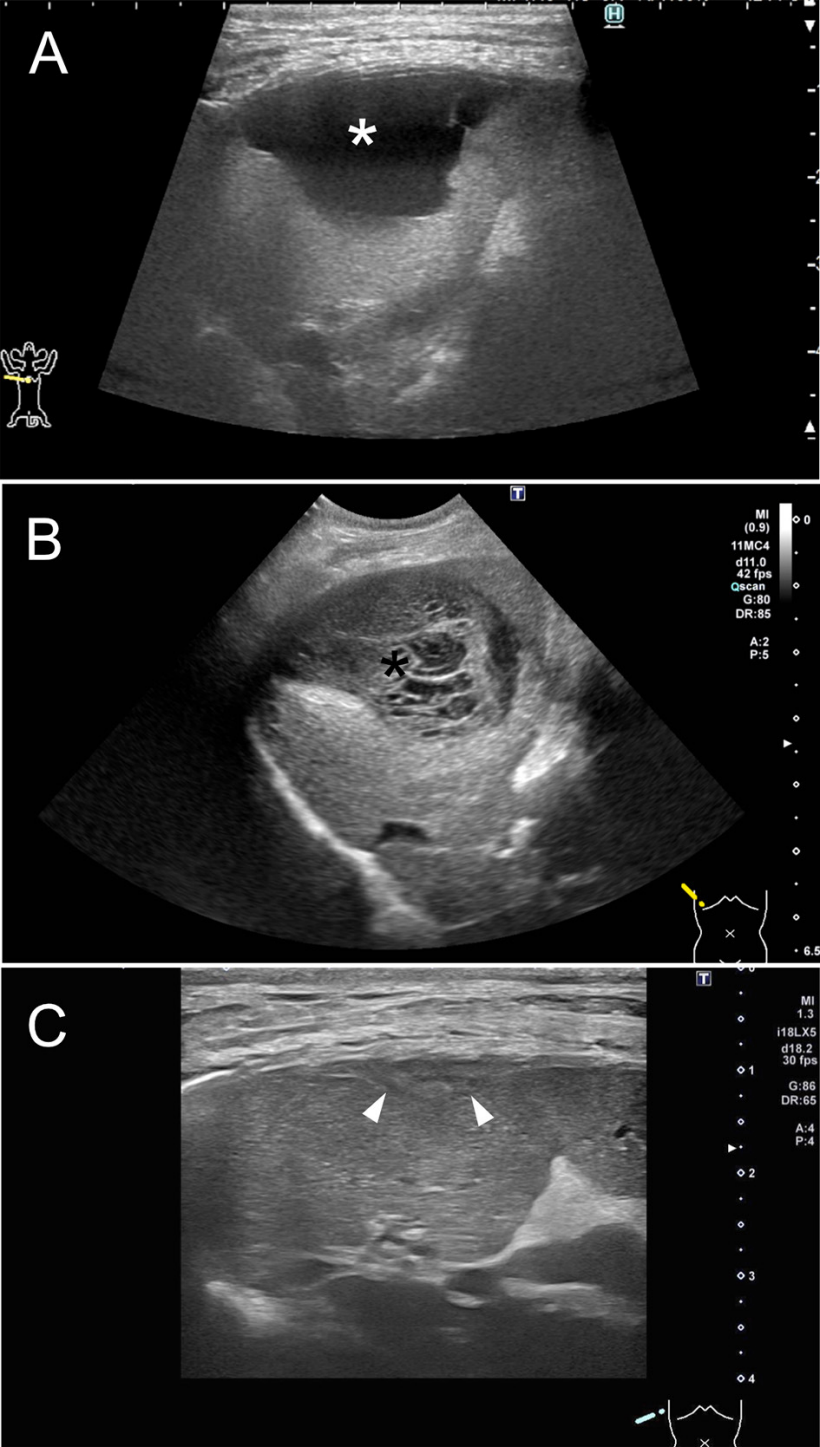
Ultrasonography of the right liver lobes through a right intercostal approach from three different time points. **(A)** On initial presentation, well-defined anechoic localized fluid accumulation was detected around the right liver lobes. **(B)** On day 6, ultrasonography revealed that the size of biloma was stable, but its appearance had changed, showing a decreased fluid and organized echoic non-mobile collections, such as mucocele (black asterisk). **(C)** On postoperative day 10, abdominal ultrasonography was able to detect a trace level of biloma wall (white arrow-head) but no fluid accumulation around the right hepatic division.

Abdominal ultrasonographic examination revealed enlargement of the gallbladder, which showed a kiwi-like pattern and stella combination with residual central echogenic bile, indicating gallbladder mucocele (Figure 2A). No defect in the gallbladder wall was identified. Although common biliary duct dilation was observed (4.4 mm), no echogenic sediment or debris within the common biliary duct was observed (Figure 2B). A small amount of ascites was detected around the spleen and bladder (Figure 2C). Through the right intercostal approach, well-defined anechoic localized fluid accumulation was detected around the right liver lobes (Figure 3A). The amount of accumulated fluid was much larger compared to the ascites. No abnormality was found in other organs.

The localized fluid in the space within the right hepatic division was collected through abdominocentesis *via* the right intercostal space of the 10th−11th thoracic vertebra before CT examination and subsequently analyzed. The laboratory evaluation of fluid showed that it comparable to an exudate, with a total nucleated cell count of 11,500/μl and protein of 3.9 g/dl. Cytological examination revealed neutrophilia, despite the absence of pyogenic bacteria. The fluid accumulating around the right liver lobes had a bilirubin concentration of 11.4 mg/dl, which was more than twice as high as that in serum, and its glucose concentration was high compared to that in serum. Ascites could not be collected because of a small volume of effusion. The dog was diagnosed with gallbladder mucocele, and the localized bile fluid accumulation indicated the possibility of biloma around the right hepatic division.

To evaluate the bile leakage in the right hepatic division, DIC-CT was performed in accordance with previous studies (12, 13). A 22-gage over-the-needle catheter was inserted into the cephalic vein of the dog, and a dose of 100 mg iodine/kg meglumine iotroxate (Biliscopin; Bayer Schering Pharma, Berlin, Germany) was administered over 30 min. After administering the contrast agent, general anesthesia was induced using propofol (7 mg/kg, to effect intravenously) and maintained with isoflurane (minimum alveolar concentration of 1.3–1.5%) at 2 L/min of oxygen. CT images were obtained using an 80-slice CT scanner (Aquilion PRIME; Canon Medical Systems, Otawara, Tochigi). Initially, the dog was positioned and scanned in the ventral recumbent position with the following scanning parameters: X-ray tube potential of 80 kV, X-ray tube current of 200 mA, slice thickness of 0.5 mm, reconstruction interval of 0.5 mm, tube rotation time of 0.5 s, and helical detector pitch of 0.813. Apnea was induced during CT scans using a stop ventilator.

**Figure 4:**
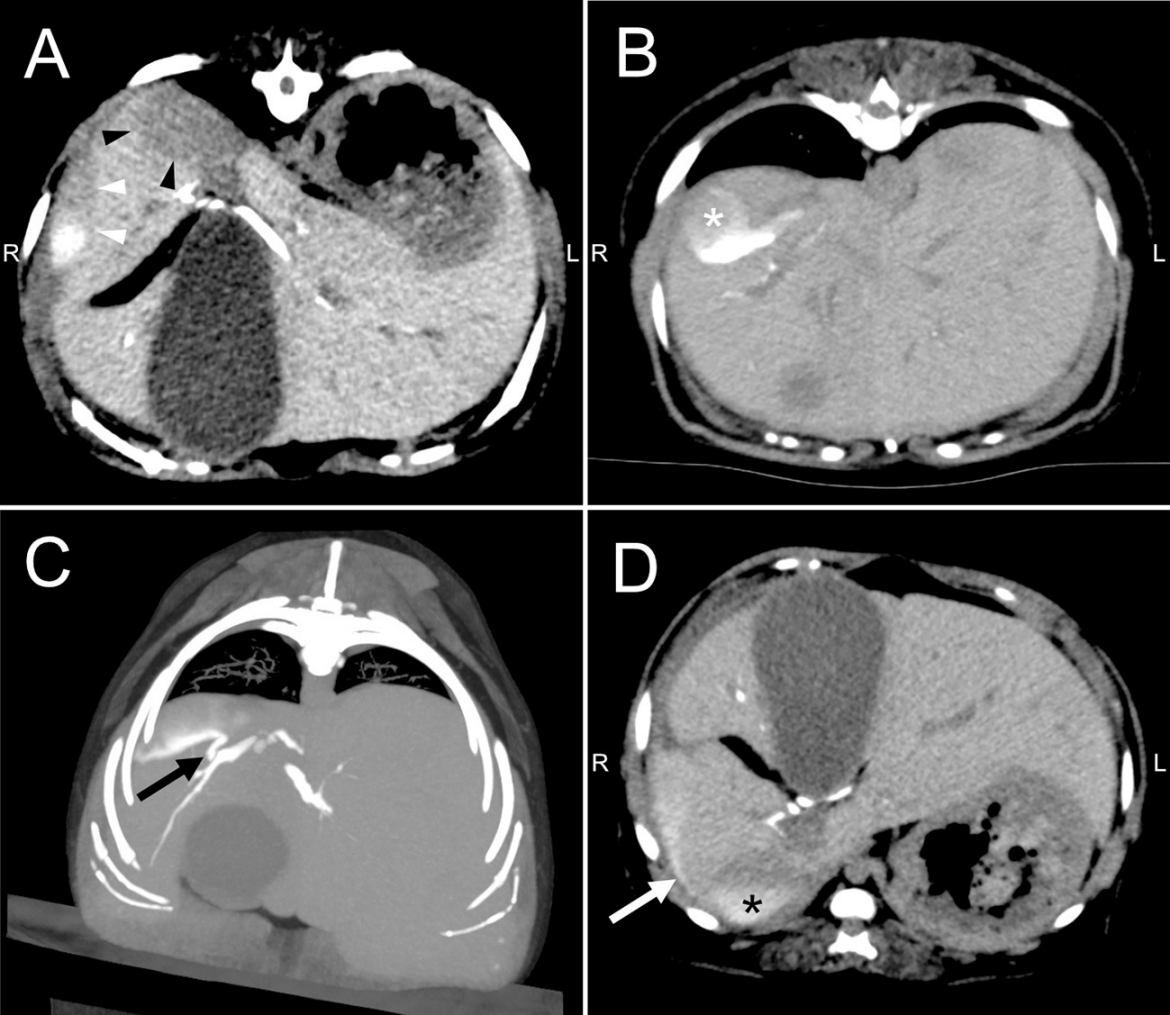
Drip infusion cholecystocholangiography with computed tomography of the abdomen in a dog with biloma. **(A)** Transverse views revealed two well-defined large fluid collections, where one is in between right medial and lateral lobe (white arrow-head) and the other in between the right lateral lobe and caudate process of caudate lobe (black arrow-head). **(B)** The CT images showed that fluid collection was enhanced by the contrast agent (white asterisk) in the ventral recumbent position. **(C)** The reconstruction image showed communication between the fluid collection and intrahepatic bile duct of right lateral lobe (black arrow). **(D)** Drip infusion cholecystocholangiography with computed tomography images after repositioning to dorsal recumbency showed that the leaked contrast agent fell dorsally via gravity (white arrow) and confirmed communication between each fluid collections (black asterisk).

On DIC-CT in the ventral recumbent position, the contrast agent filled the intra and extrahepatic bile ducts, common bile duct, and duodenum, except for the gallbladder. The CT images showed two well-defined large fluid collections (Figure 4A): one between right medial and lateral lobe (3.6 × 1.7 × 2.4 cm) and the other between the right lateral lobe and caudate process of caudate lobe (3.0 × 0.9 × 3.2 cm). Three-dimensional DIC-CT views revealed that the former was enhanced by the contrast agent and that it communicated with an intrahepatic bile duct of the right lateral lobe (Figures 4B,C). The dog was then repositioned in dorsal recumbency and rescanned to evaluate the displacement of contrast agent. Accordingly, the images showed that the leaked contrast agent fell dorsally through gravity (Figure 4D), and the DIC-CT images confirmed communication with each fluid collections. The dog showed no side reaction during and after DIC-CT. Thereafter, CT angiography using an intravenous injection of 600 mg iodine/kg iohexol (Omnipaque 300, GE Healthcare, Oslom Noeway) was performed for anatomical evaluation and surgical planning. From these findings, the dog was diagnosed with biloma caused by a leakage of the intrahepatic bile duct in the right lateral lobe.

We presumed that cholecystectomy was appropriate for the prevention of gallbladder rupture. Additionally, the right lateral hepatic lobectomy was considered for the treatment of bile leakage. However, conservative therapy with intravenous crystalloid fluid therapy, low molecular weight heparin (150 IU/kg as an intravenous continuous infusion for 24 h), and ampicillin (20 mg/kg, intravenous every 12 h) was performed before surgery due to following reasons: having mild clinical signs due to localized bile peritonitis and expecting the adhesion of intrahepatic bile duct, which may prevent lobectomy.

**Figure 5:**
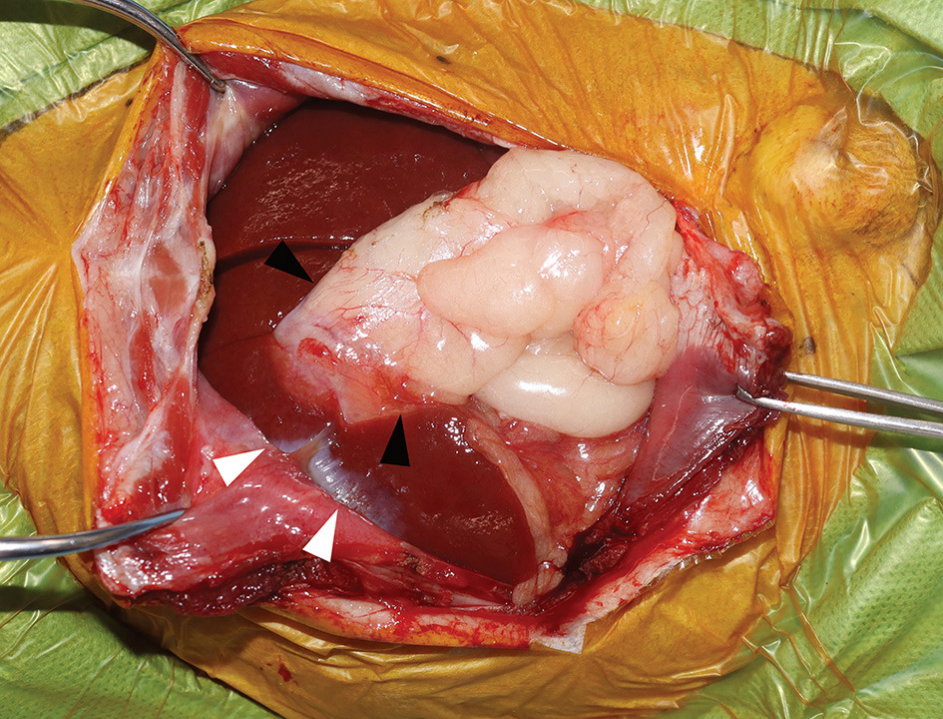
Intraoperative findings. Image showing severe adhesion between the right medial lobe of the liver and the abdominal wall (white arrow-head) and between the gallbladder and omental fat (black arrow-head).

On day 6, ultrasonography revealed that the size of biloma was stable, but its appearance had changed such that the fluid decreased and organized echoic non-mobile collections, such as a mucocele, appeared (Figure 3B). Exploratory laparotomy was performed through the ventral midline approach with right paracostal incision. Exploration of the abdomen revealed severe adhesion between the right medial lobe of the liver and the abdominal wall, as well as between the gallbladder and omental fat (Figure 5). Hepatic lobectomy was not performed due to the decrease in the amount of fluid within the biloma and the severe adhesion sealing intraoperatively. After cholecystectomy and liver biopsy, catheterization, and flushing of the common biliary duct was performed to confirm duct patency. The dog's recovery from general anesthesia was uneventful.

**Figure 6:**
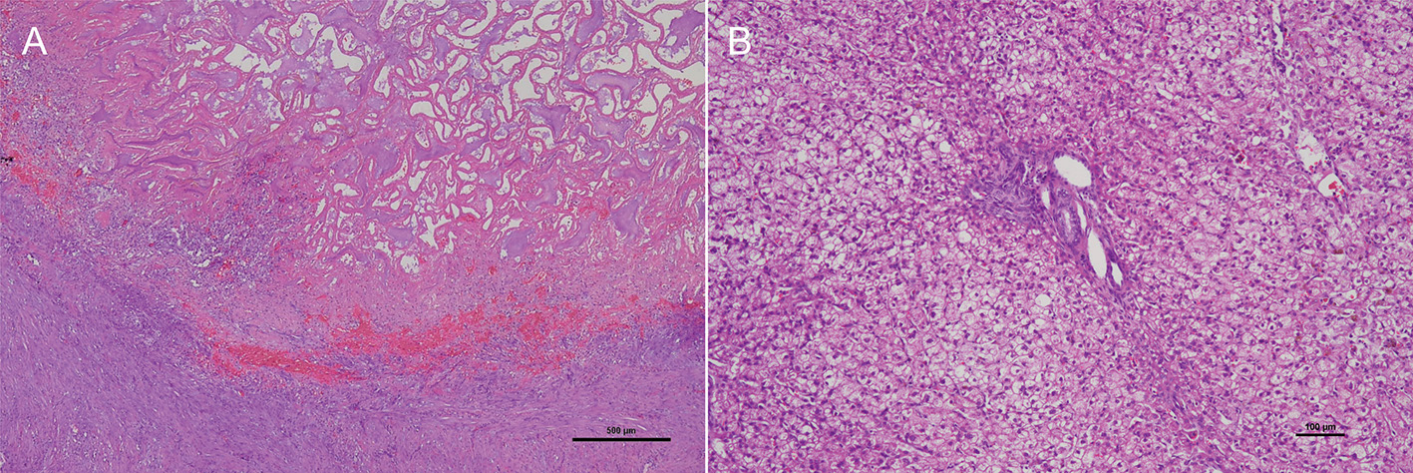
Photomicrography of the hematoxylin and eosin-stained section of the gallbladder and liver. **(A)** A large viscous accumulation of mucus that filled and distended the gallbladder lumen and full thickness necrosis of the gallbladder wall. **(B)** Liver biopsy specimens revealed cytoplasmic vacuolation of hepatocytes without any inflammation and fibrosis of the hepatic parenchyma.

Tissues of the gallbladder and liver were submitted for histopathological analysis. Despite observing a large viscous accumulation of mucus that filled and distended the gallbladder lumen and full thickness necrosis of the gallbladder wall (Figure 6A), no findings of rupture were noted. Liver biopsy specimens revealed cytoplasmic vacuolation of hepatocytes without any inflammation and fibrosis of the hepatic parenchyma (Figure 6B). The dog was diagnosed with gallbladder mucocele with infarctions of the gallbladder and vacuolar hepatopathies.

Seven days after the surgery, the dog was in good condition and discharged from the hospital on the same day. The dog subsequently visited our hospital on postoperative day 10 for the removal of the skin sutures, during which abdominal ultrasonography detected a trace level of biloma wall and no fluid accumulation around the right hepatic division (Figure 3C). Seven months after the surgery, the dog was doing well without any recurrence of clinical signs determined via telephone contact with the owner.

## Discussion

We herein detail the experience with a rare case of biloma due to a leakage from intrahepatic bile duct in the right lateral lobe. To the best of our knowledge, this has been the first report describing the usefulness of DIC-CT for the detection of the bile leakage site within the intrahepatic duct in veterinary medicine.

A few reports have been available on bilomas in veterinary medicine, including three dogs and one cat with biloma. In one dog, biloma developed after surgical treatment of intrahepatic portosystemic shunt (7), whereas in the other dog, biloma was associated with trauma (hit by a car) (10). Moreover, biloma in a cat developed after open liver biopsy (9). Although one dog had also been diagnosed with biloma 12 days after cholecystectomy, this report has not been discussed during the causes of biloma (8). The current case had no history of biliary surgery, liver biopsy, or abdominal trauma, suggesting an unknown cause of biloma. Previous studies showed that the cause of bilomas in humans included spontaneous leakage of the intrahepatic bile duct (3, 4). The current dog may have developed biloma spontaneously. However, considering the histopathological diagnosis of gallbladder mucocele, the leakage of the intrahepatic duct in the right lateral lobe may have been caused by biliary obstruction due to the high viscosity of its contents.

DIC-CT provided valuable information regarding the localization of the bile leakage. This imaging technique has been used to assess the bile duct system in human patients, and its usefulness has also been recently reported in dogs with gallbladder mucocele and cats with cholelithiasis (12, 13). Nonetheless, the mentioned studies have focused on the evaluation of bile duct patency or the detection of extrahepatic bile duct obstruction secondary to its highly viscous contents or cholelithiasis. Dogs and cats with extrahepatic bile duct obstruction usually undergo cholecystectomy as well as intraoperative catheterization and flushing of the common bile duct (14). Conversely, hepatic lobectomy has been the treatment option for a leakage from intrahepatic bile duct provided that persistent bile leakage or major biliary injuries occur (15). However, hepatic lobectomy is highly invasive and carries increased risk of surgical complications, although it depends on the affected region of liver, especially in cases involving the right division of the liver as in our dog. Therefore, we performed DIC-CT to determine the bile leakage site given that this information was needed to establish a treatment plan and surgical approach. DIC-CT may be a useful method for evaluating bile leakage as well as bile duct patency.

Biliary rupture and subsequent bile peritonitis can be a clinically mild condition, or it can be a life-threatening condition (16, 17). Our dog presented with only mild gastrointestinal signs and a mild increase in C-reactive protein levels (3.75 mg/dl) at presentation. Bilomas are encapsulated collections of bile; hence, it is assumed that only a limited inflammatory response occurred in other organs.

The most common therapeutic approach in human patients with bilomas is percutaneous drainage (3, 4). Studies in veterinary medicine involving two dogs with bilomas who underwent percutaneous drainage showed that both dogs had good outcomes without such a surgical approach for biloma (8, 10). However, the current dog with biloma had been managed with supportive therapy and cholecystectomy. Without adequate medical management, the size or number of bilomas could have increased, and the clinical signs could have worsened, possibly necessitating aggressive drainage, or surgical treatment (18, 19).

A review of the literature on the use of iotroxate in 2,492 patients had shown that adverse reactions (e.g., anaphylaxis, urticaria, and respiratory distress) do occur in human patients following DIC-CT using meglumine iotroxate (20), but at relatively low rates (3.5%) (21). Additionally, previous studies have suggested that the rate of adverse reactions was three times lower with infusions at a constant rate than with bolus injections (21). Therefore, studies have proposed that the tolerance of intravenous biliary contrast agent can be improved when a slow infusion technique is used in humans (22, 23). Previously reported DIC-CT studies in dogs and cats had also used constant rate infusions over 30 min, with no significant side effects having been reported (12, 13). The current dog also did not show side effects after meglumine iotroxate infusion.

Overall, this case report showed that DIC-CT may be a useful method for detecting the bile leakage site within the intrahepatic duct. Further studies including a larger number of patients are needed to elucidate the usefulness and potential adverse reactions associated with DIC-CT.

## Data Availability Statement

The original contributions presented in the study are included in the article/supplementary material, further inquiries can be directed to the corresponding author/s.

## Ethics Statement

The animal study was reviewed and approved by Hokkaido University Veterinary Teaching Hospital. Written informed consent was obtained from the owners for the participation of their animals in this study.

## Author Contributions

MT drafted this article and all co-authors carefully revised it. All authors contributed the management of this case, the conception of the work, and interpretation of data. All authors contributed to the article and approved the submitted version.

## Funding

This open access publication fee was supported in part by funds of Rakuno Gakuen University.

## Conflict of Interest

The authors declare that the research was conducted in the absence of any commercial or financial relationships that could be construed as a potential conflict of interest.

## Publisher's Note

All claims expressed in this article are solely those of the authors and do not necessarily represent those of their affiliated organizations, or those of the publisher, the editors and the reviewers. Any product that may be evaluated in this article, or claim that may be made by its manufacturer, is not guaranteed or endorsed by the publisher.
